# Nanomaterials for stroke diagnosis and treatment

**DOI:** 10.1016/j.isci.2024.111112

**Published:** 2024-10-09

**Authors:** Yang Liu, Junying Li, Huaijuan Guo, Chao Fang, Qiaoling Yang, Wen Qin, Hai Wang, Yong Xian, Xuebing Yan, Binxu Yin, Kun Zhang

**Affiliations:** 1 Department of Oncology, The Affiliated Hospital of Yangzhou University, Yangzhou University, Yangzhou, Jiangsu, China; 2 Department of Pharmacy and Central Laboratory, Sichuan Academy of Medical Sciences, Sichuan Provincial People’s Hospital, University of Electronic Science and Technology of China, No. 32, West Second Section, First Ring Road, Chengdu 610072, China; 3 Instrumentation and Service Center for Science and Technology, Beijing Normal University, No. 18 Jinfeng Road, Zhuhai 519087, Guangdong Province, China; 4 Jiangsu Provincial Innovation and Practice Base for Postdoctors, Suining People’s Hospital, Affiliated Hospital of Xuzhou Medical University, Suining 221200, China

**Keywords:** Diagnostics, Therapeutics, Nanomaterials, Biomedical materials

## Abstract

Nanomaterials and nanotechnology innovations possess unique physicochemical properties that present new opportunities in the realm of stroke detection, diagnosis, and treatment. This comprehensive review explores the utilization of nanomaterials in the diagnosis and treatment of strokes, encompassing recent advancements in computed tomography (CT), magnetic resonance imaging (MRI) and magnetic particle imaging (MPI), as well as groundbreaking applications of nanomaterials and bionanomaterials in drug delivery systems and brain tissue repair. Additionally, this review meticulously examines significant challenges such as biocompatibility toxicity and long-term safety, proposing potential strategies to surmount these obstacles. Moreover, this review delves into the application of nanomaterials to improve the clinical diagnosis of stroke patients, elucidates the potential of nanotechnology in post-stroke therapy, and identifies future research directions and potential clinical applications.

## Graphical abstract

**Figure:**
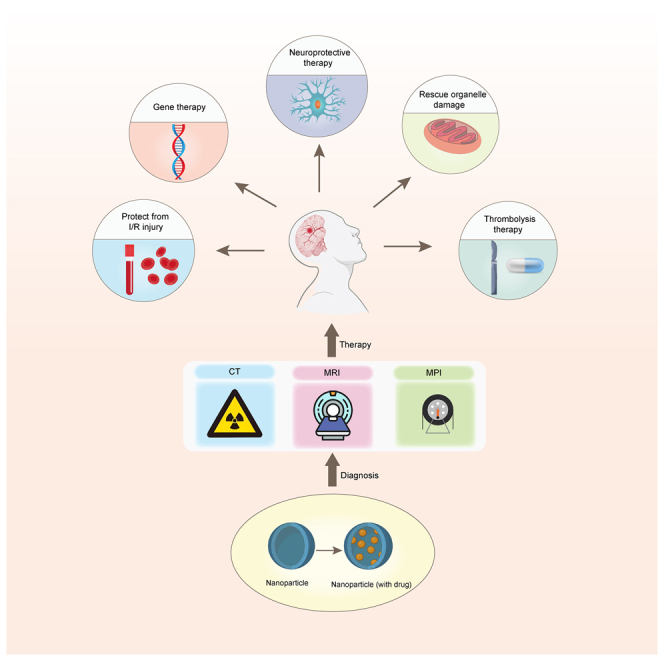

## Introduction

Stroke is a significant global cause of death and disability, posing a serious health risk to populations. The World Stroke Organization – Lancet Neurology Commission Report predicts that stroke-related fatalities will increase by 50% and stroke-related disabilities will rise by 30% by 2050.^1^ China, in particular, bears a high burden of stroke and is confronted with a substantial stroke challenge.^2^^,^^3^

Stroke is a clinical syndrome characterized by the abrupt onset of a focal neurologic deficit caused by vascular injury, such as infarction or hemorrhage, to the central nervous system. Ischemic strokes, accounting for 85% of all strokes, typically result from atherosclerosis of small vessels, cardiac embolism, or atherosclerotic thromboembolism of large arteries. The remaining 15% of strokes occur due to intracranial bleeding, which can affect various regions of the brain including the basal ganglia, brainstem, cerebellum, or lobes.^4^ Ischemic stroke and cerebral infarction cause neuronal damage, which can be categorized into three main types based on their underlying pathogenesis: direct neuronal damage resulting from ischemia and infarction, neuronal damage caused by oxidative stress due to excessive production of reactive oxygen species (ROS) triggered by ischemia-induced vascular occlusion, and neuronal damage caused by ischemia-induced inflammation.^5^^,^^6^^,^^7^

Accurate diagnosis is essential to optimize stroke treatment.^8^ Clinicians initially conduct a rapid history, inquiring about the onset and nature of symptoms from the patient or a knowledgeable informant. Subsequently, a physical examination, inclusive of a neurological assessment, is promptly conducted to identify potentially affected brain regions.^9^ Various neurological rating scales, such as the National Institutes of Health Stroke Scale (NIHSS), are employed to assess stroke severity and monitor the response to treatment.^10^ Imaging studies are then initiated, with cranial CT scans as the preferred method due to their rapid ability to differentiate between ischemic and hemorrhagic strokes. MRI, specifically diffusion-weighted imaging (DWI), is highly sensitive for detecting acute cerebral infarction and significantly enhances the accuracy and speed of stroke diagnosis, enabling swift differentiation between ischemic and hemorrhagic strokes and facilitating timely treatment initiation.^11^ In addition, digital subtraction angiography (DSA) provides detailed vascular images essential for supporting diagnostic and therapeutic decisions.^12^ The time-sensitive nature of stroke diagnosis and treatment poses a significant challenge. The integration of nanotechnology into stroke diagnosis and treatment offers an innovative approach that has the potential to significantly improve patient outcomes.

Thrombolytic therapy is currently the most commonly used treatment for ischemic stroke and cerebral infarction. This therapy involves the use of thrombolytic drugs and mechanical devices to restore blood flow in the cerebral arteries, ultimately leading to the recovery of brain tissues and neurological functions.^13^^,^^14^ According to the WSO, intravenous alteplase should be administered to patients with symptoms of ischemic stroke within 4.5 h and without contraindications to intravenous thrombolysis. Additionally, mechanical thrombectomy should be administered to patients with acute stroke and occlusion of large arteries in the anterior circulation within 6 h of symptomatic onset.^15^ It is crucial to administer intravenous thrombolysis in conjunction with mechanical thrombectomy and not delay the latter for the former.^16^

Both intravenous thrombolysis and mechanical thrombectomy have their limitations. Intravenous thrombolysis is restricted by a short time window for treatment and presents higher risks for patients with cerebral hemorrhage. On the other hand, mechanical thrombectomy can only be conducted within a wider time frame and applies solely to patients with larger, anatomically more proximal vessel occlusions, necessitating sophisticated operational technical support. To address these challenges, the integration of new technologies is urgently required, and nanotechnology may present a promising breakthrough. In recent years, nanotechnology has been increasingly utilized in stroke therapy, employing nanoparticles with diverse properties for stroke diagnosis and treatment. This article discusses the development of multifunctional thrombolytic drugs, which not only encompass thrombolytic agents but also include anti-inflammatory, antioxidant, neuro/vascular protective agents, and imaging agents.^17^ These nanomaterials possess unique characteristics, such as size effect and surface effect, and exhibit distinctive behavior within biological systems, thus demonstrating their significant potential in targeted drug delivery, disease monitoring, and diagnosis.

**Figure 1:**
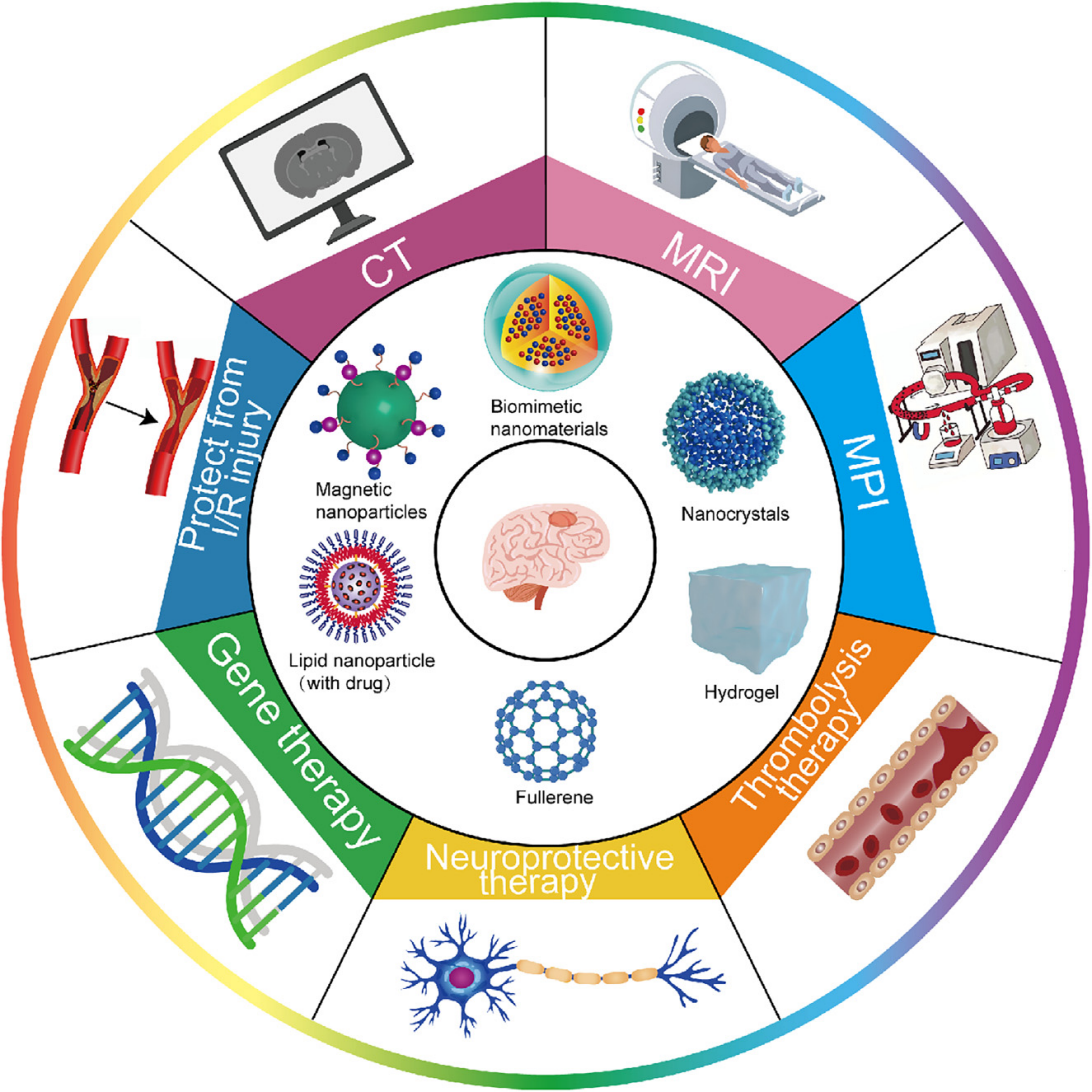
The innovative integration of nanomaterials and nanotechnology in imaging modalities such as computed tomography (CT), magnetic resonance imaging (MRI) and magnetic particle imaging (MPI) has greatly improved the diagnostic accuracy and reliability of early stroke detection They have also introduced novel therapeutic concepts and protocols for stroke treatment, especially in thrombolysis, neuroprotective and gene therapy, and protection from ischemia-reperfusion (I/R) injury.

We summarize recent research advances in the use of nanomaterials for stroke diagnosis and treatment (Figure 1). It covers various topics, including diagnostic and therapeutic applications, as well as future challenges (Table 1). The detailed description focuses on how nanomaterials have the potential to improve the accuracy and speed of early disease recognition. Additionally, we explore innovative applications of nanotechnology in stroke treatment, particularly drug delivery systems and the repair of damaged brain tissue. The discussion also highlights the potential benefits of these technologies in improving treatment outcomes and patient recovery. Furthermore, we examine the primary obstacles to implementing nanomaterials in this field, such as biocompatibility, toxicity, and long-term safety concerns, and propose potential solutions to address these challenges. Finally, we discuss the potential of nanomaterials to enhance the clinical prognosis of stroke patients through novel diagnostic and therapeutic approaches. This review offers fresh perspectives and opportunities for future research and clinical practices, while also serving as a theoretical foundation and reference framework for further nanotechnology research in the field of stroke.

**Table 1:** Applications of nanomaterials in stroke

Nanomaterials	Approach	Mechanism	References
PEGylated BaHoF5 nanoparticles (NPs)	CFA and CTP imaging	It can replace iodine-based CM for diagnostic contrast-enhanced imaging of patients with kidney/heart diseases	Wang et al.^18^
PEGylated supermagnetic iron oxide nanoparticles (SPIONs)	MRI	PEGylated SPIONs to highlight BBB damage by MRI	Liu et al.^19^
BSA–MnO2 nanoparticles (BM NPs)	MRI	The BM NPs showed a high T1 relaxivity (r1 = 5.9 mM−1 s−1), remarkable MR imaging ability, and good biocompatibility, allowing the noninvasive timely visualization of BBB permeability	Hou et al.^20^
MnCO3@BSA-ICG NPs	MRI	MnCO3@BSA-ICG NPs quickly and efficiently led to the MR/PA contrast enhancements in the infarcted area while not in the normal region, allowing a timely and accurate diagnosis of AIS.	Song et al.^21^
Fe_3_O_4_	MRI	Construction of low immunogenicity nanoprobes by covalently attaching self-peptides to biocompatible Fe3O4 nanoparticles as a stealth coating	Zhang et al.^22^
PAMNs	MRI	PAMNs loaded with L-arginine and γ-Fe_2_O_3_ magnetic nanoparticles can specifically target to different types of damaged blood vasculature.	Li et al.^23^
superparamagnetic iron oxide nanoparticles	MPI	Superparamagnetic iron oxide nanoparticles can be used as a contrast agent for MPI to assess cerebral perfusion and vascular anatomy in seconds.	Ludewig et al.^24^
SiO_2_-MNP	Thrombolysis Therapy	Biocompatible SiO2-MNP can be used as a magnetic targeted drug carrier for improved clinical thrombolytic therapy.	Chen et al.^25^
SPIONs	Thrombolysis Therapy	SPIONs loaded with tPA are able to achieve targeted drug delivery into gels that mimic thrombi using external magnets for precise guidance, thereby increasing thrombolytic efficiency and reducing potential damage to surrounding healthy tissue.	Heid et al.^26^
cRGD urokinase liposomes.	Thrombolysis Therapy	cRGD liposomes could significantly reduce the dose of urokinase by 75% while achieving the equivalent thrombolysis effect as the free urokinase	Zhang et al.^27^
nanoplatelets	Thrombolysis Therapy	The nanoplatelets exhibit improved therapeutic efficacy over free rt-PA in an ischemic stroke model.	Xu et al.^28^
PEG-uPA-PEGPPG-PEG	Thrombolysis Therapy	The biological activity of uPA is masked under normal conditions, which can reduce the risk of acute bleeding complications, and the recovery of protein activity at acidic pH and 33°C is conducive to mild hypothermia thrombolytic therapy	Jin et al.^29^
DNA thrombolytic nanomachine	Thrombolysis Therapy	The DNA thrombolytic nanomachine is capable of detecting thrombin, a biomarker for blood clots, in complex pathophysiological conditions within the blood vessels.	Yin et al.^30^
Exosomes	Neuroprotective Therapy	NSCs could produce hypoxic exosomes for efficient treatment of ischemic stroke.	Jiang et al.^31^
BDNF- hnsc - exo	Neuroprotective Therapy	BDNF-hNSC-Exo not only inhibited the activation of microglia, but also promoted the differentiation of endogenous NSCs into neurons.	Zhu et al.^32^
YC-1@[RBC-PL] NVs	Neuroprotective Therapy	It reduces ischemic damage to the NVU by decreasing infarct volume, preserving the integrity of the BBB, and inhibiting the activation of astrocytes and microglia.	Liu et al.^33^
SDF-1-EVs	Neuroprotective Therapy	SDF-1-EVs were found to significantly enhance NSC migration and differentiation into neurons, while also reducing brain atrophy volume.	Ruan et al.^34^
C_60_-Arg	Neuroprotective Therapy	It can cross the blood-brain barrier and release nitric oxide, which is derived from the decomposition of L-arginine residues, thereby promoting vasodilation 63.	Kukaliia et al.^35^
GB-NSSPNE	Neuroprotective Therapy	It regulates the release and enhances cellular uptake, resulting in a significantly improved permeability of GB. This leads to a notable increase in the oral bioavailability of GB, thereby enhancing its neuroprotective effect	Liu et al.^36^
SHp-NM@Edv/RCD	Protecting the Brain from I/R injury	The drug Edv is precisely released at the site of cerebral ischemia/reperfusion injury by the controlled release device (RCD) enclosed within the SNM-NPs.	Dong et al.^37^
EDV/Cur/NapFFY	Protecting the Brain from I/R injury	Animal studies have revealed the EDV/Cur/NapFFY hydrogel’s ability to effectively enhance brain plasticity and promote functional recovery in a photothrombotic mouse model.	Jia et al.^38^
PVAX	Protecting the Brain from I/R injury	Upon degradation of the PVAX nanoparticles, VA is released, which effectively reduces the production of ROS and exhibits both anti-inflammatory and anti-apoptotic effects.	Lee et al.^39^
Ti_2_C@BSA-ISO	Protecting the Brain from I/R injury	Ti2C@BSA-ISO showed a robust capacity for scavenging free radicals.	Fan et al.^40^
NBP-loaded SON	Protecting the Brain from I/R injury	It aims to reduce endothelial permeability induced by ischemia/reperfusion, improve dendritic remodeling, and enhance synaptic plasticity of neurons in the injured brain tissue.	Yang et al.^41^
PEG-Acetal-PCL-PEG	Protecting the Brain from I/R injury	It modified with cRGD and TPP on the long and short PEG chains, respectively, effectively alleviates oxidative stress and inflammation.	Wang et al.^42^
pDA-MNOF	Protecting the Brain from I/R injury	pDA-MNOF nanoparticles enhance neuronal HO1 and SOD2 via STAT3, protecting against oxidative stress, reducing infarct size, and promoting behavioral recovery through free radical scavenging and pro-angiogenesis.	Wang et al.^43^
a pH-triggered polymersome nanoplatform	Protecting the Brain from I/R injury	pH-triggered polymer nanoplatform delivers DNase 1 to the brain, mitigating thrombotic inflammation in ischemic stroke by disintegrating in acidic conditions, scavenging NETs, and inhibiting platelet activation and microthrombosis.	Li et al.^44^
Pic@AR5	Protecting the Brain from I/R injury	Pic@AR5 nanoparticles impede neutrophil adhesion and BBB infiltration, reducing neuroinflammation by inhibiting Syk signaling in ischemic regions	Song et al.^45^
NTA/Ce^4+^/C-176	Protecting the Brain from I/R injury	Nanoparticles deliver C-176 and Ce4+ to counteract chronic inflammation caused by dsDNA fragments activating the cGAS-STING pathway in microglia.	Zhu et al.^46^
Drug-Free Biomimetic Nanovehicle	Protecting the Brain from I/R injury	The nanovehicle consists of polyfluorocarbon and Pluronic P123; the former delivers oxygen, while the latter inhibits MMP-9 to protect the blood-brain barrier. After intravenous injection, it targets cerebral ischemic lesions, increasing tolerance to ischemia and resistance to reperfusion.	He et al.^47^
OMV@PGZ	Protecting the Brain from I/R injury	It encapsulates pioglitazone (PGZ) nanoparticles within outer membrane vesicles, crosses the blood-brain barrier, releases PGZ in ischemic areas, suppresses ferroptosis, and reduces inflammation.	Pan et al.^48^
T-TMP	Protecting the Brain from I/R injury	Another study showed that modifying the T-TMP surface with the CFLFLF peptide allows it to bind to neutrophils and use their migratory ability to target ischemic brain regions.	Mu et al.^49^
MPO	Gene therapy	MPO targets brain microvascular endothelial cells, promoting their migration and tube formation. *In vivo*, MPO reduces cerebral infarction, increases capillary density, and promotes microvessel growth by upregulating Vegfa and Vegfr2.	Shen et al.^50^
B-PDEA	Gene therapy	B-PDEA is a ROS-responsive charge-reversal polymer vector that efficiently mediates gene transfection in NSCs and induces BDNF expression for the treatment of ischemic stroke.	Jiang et al.^51^
PLGA	Gene therapy	PLGA nanoparticles encapsulated with GFAP:SOX9:tdTOM reduce ischemia-induced neurological deficits and infarct volume via the prostaglandin D2 pathway.	Shin et al.^52^
ZnMNPs	Gene therapy	Magnetic nanoparticle therapy for stroke controls stem cell migration with a magnetic field, boosting neuronal differentiation and neurotrophic factor secretion to repair and restore damaged brain tissue.	Yun et al.^53^
CPTK@PMH nanoerythrocyte	Tissue Engineering	The CPTK@PMH nanoerythrocyte targets microthrombi, adapts to the metabolic environment of the brain, releases oxygen during ischemia, blocks ROS during reperfusion, and improves glucose metabolism and BBB protection, providing a comprehensive treatment for AIS.	Liu et al.^54^
PP@PCL	Drug Delivery	A nanocarrier combining 4T1 breast cancer and platelet membranes was developed to encapsulate paeoniflorin and poly (methyl caprolactone) liposomes (PP@PCL). It effectively inhibits neuroinflammation, enhances cerebral vascular density, promotes neurovascular regeneration, and remodels the ischemic microenvironment.	Tang et al.^55^
MFION	Rescuing Organelles	MFION boosts BDNF secretion and CXCR4 expression, thereby enhancing the homing ability of mesenchymal stem cells (MSCs) and their therapeutic efficacy in ischemic brain tissue.	Zhang et al.^56^
PATRC	Promoting Angiogenesis	PATRC successfully crossed the BBB, reduced brain infarct volume, and enhanced microvascular regeneration in the affected area.	Shen et al.^57^
PHSRN	Promoting Angiogenesis	PHSRN and SAG enhance angiogenesis, strengthen the blood-brain barrier, and improve neuroplasticity and neurological recovery.	Yang et al.^58^

### Application and advantages of nanomaterials in medicine

Nanomedicine is an emerging field in medicine that focuses on studying biological phenomena and developing advanced medical treatments using nanoscale interventions and preventive measures.^59^^,^^60^ It offers an effective approach to tackling diagnostic, therapeutic, and critical clinical challenges within biological systems.^61^^,^^62^ Of particular interest are smart nanomedicines, which have garnered considerable attention for their capacity to specifically respond to both external and internal microenvironments, thereby demonstrating highly controllable and efficient properties for treating diverse diseases.^63^^,^^64^

Various types of nanostructures have been effectively fabricated, including lipid-based components such as liposomes and lipid-based carriers, polymeric structures like micelles, dendrimers, and nanogels, and inorganic-based configurations such as metals, superparamagnetic metal oxides, and quantum dots. These nanostructures have the potential to serve as vehicles for drug delivery and encapsulation, enabling targeted drug release at specific locations.^65^^,^^66^

### Nanomaterials in stroke diagnosis

CT and MRI are the main imaging techniques used in the diagnosis of strokes because of their ability to provide accurate brain images that help in locating and evaluating the severity of the stroke. However, these methods can be expensive and not easily accessible in some healthcare settings. CT scans, although effective, expose patients to ionizing radiation, while MRI scans can be time-consuming and inappropriate for patients with certain medical conditions or implants.^67^^,^^68^

#### Computed tomography

In the field of cerebrovascular diagnostics, the detection of ischemic strokes using non-contrast CT imaging poses a significant challenge. This is primarily because there is not enough contrast differentiation between thrombi and the surrounding blood. The density of the thrombotic material is similar to that of nearby circulatory elements, making it difficult for standard imaging to provide sufficient resolution. Consequently, diagnosing ischemic episodes and their neurological manifestations in a clinical setting becomes complex.^69^

Langheinrich et al. conducted a study on cerebral ischemia in rats using micro- and nano-CT imaging. They compared two models of permanent middle cerebral artery occlusion and demonstrated the detailed visualization of the cerebral vasculature that can be achieved through these advanced imaging techniques. This research introduces a novel approach to evaluating models of cerebral ischemia.^70^^,^^71^ Building upon this technology, Wang et al. investigated the encapsulation of CT contrast agents in nanocarriers. They specifically highlighted the use of PEGylated BaHoF5 nanoprobes, which have proven effective in targeting pathological cerebral sites and overcoming physiological barriers to provide localized enhancement. These nanotechnology-enhanced probes have been validated in CT angiographic and perfusion studies. They offer high-contrast visualization of cerebral abnormalities with reduced doses compared to conventional iodinated contrast agents, thereby decreasing the potential for iatrogenic complications.^18^ The combination of nano-CT and nanoparticle-mediated contrast delivery shows promising potential for advancing neuroimaging and potentially altering the standard of care for stroke diagnosis.

#### Magnetic resonance imaging

MRI is a versatile imaging technique that plays a crucial role in diagnosing diseases, guiding treatment, and evaluating treatment efficacy. To enhance medical images, contrast agents are used to target specific areas. However, traditional contrast agents, which mainly consist of small molecules, have limitations, including rapid metabolism and poor targeting in both human blood circulation and tumors. As a result, their clinical effectiveness is limited.^72^ Nevertheless, the use of nanomaterials holds promise for overcoming these limitations and improving the diagnostic capabilities and standards of medical imaging.^73^

**Figure 2:**
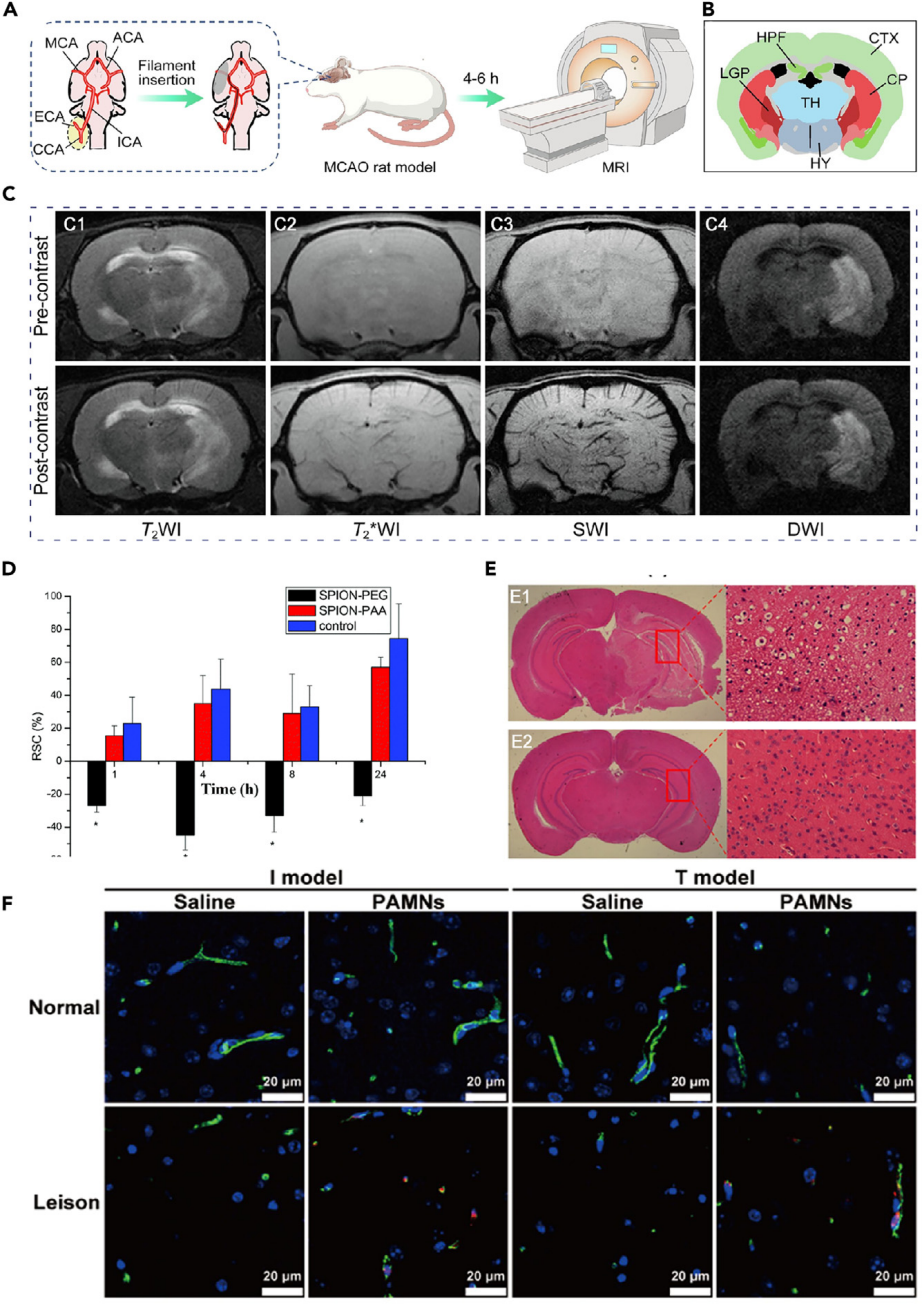
The application of nanoparticles in MRI (A) Schematic representation of the rat rMCAO model construction and the MRI study procedures. (B) Coronal brain atlas of the rat corresponding to the imaging plane. (C) (C1) T2WI, (C2) T2∗WI, (C3) SWI and (C4) DWI of rMCAO rat brain captured before (upper) and 5 min after (lower) injection of the nanoprobes (10 mg Fe per kg body weight). Reproduced with permission from ref.^22^ Copyright 2023 Materials & Design. (D) Relative changes in signal intensity across different groups (*n* = 3). ∗*p* < 0.05 compared to the SPION-PAA and saline groups. Reproduced with permission from ref.^19^ Copyright 2014 Nanoscale. (E) HE staining of brain sections of MCAO I/R and sham brain. Reproduced with permission from ref.^21^ Copyright 2022 Biochem Biophys Res Commun. (F) Simultaneous presence of brain microvessels and PAMNs, as identified by CD31 immunofluorescence labeling. Reproduced with permission from ref.^23^ Copyright 2023 Science China Materials.

In recent years, several studies have focused on MR nanocontrast agents for stroke diagnosis. One notable study conducted in 2014 by Liu et al. introduced a novel method for dynamic imaging of blood-brain barrier (BBB) damage. This approach utilized PEGylated supermagnetic iron oxide nanoparticles (SPIONs) as a contrast agent. By utilizing SPIONs, it became possible to conduct dynamic imaging of both BBB permeability changes and ischemic lesions concurrently using T2-weighted MRI (T2WI) (Figure 2D).^19^

In 2021, Hou et al. prepared BSA-MnO2 nanoparticles (BM NPs) through a simulated sterilization method. These BM NPs demonstrate high T1 relaxation (T1) (r1 = 5.9 mM-1 s-1), significant MRI capabilities, and excellent biocompatibility. Consequently, they are suitable for noninvasive and timely imaging of BBB permeability in a rat middle cerebral artery occlusion (MCAO) model.^20^ In 2022, Song et al. developed MnCO3 nanoparticles using bovine serum albumin (BSA) as a scaffold. For pH-responsive manganese (Mn)-based nanoplatform, MnCO3@BSA-ICG NPs, they integrated indocyanine green (ICG) as a photoacoustic probe (Figure 2E).^21^ The resulting nano platform exhibits rapid and efficient enhancement of magnetic resonance/photoacoustic (MR/PA) contrast in infarcted areas, enabling timely and accurate diagnosis of acute ischemic stroke (AIS).

Nanomaterials have significantly enhanced the efficacy of stroke MRI by constructing nanoprobes with low immunogenicity. Zhang et al. successfully extended the circulation time of nanoprobes in the bloodstream and effectively enhanced vascular contrast during susceptibility-weighted imaging (SWI) by covalently bonding self-peptides to the surface of biocompatible Fe_3_O_4_ nanoparticles. Combined with DWI, this technology can accurately delineate the extent of the infarct core, collateral vessels, and ischemic penumbra, providing vital information for the diagnosis and treatment of stroke (Figures 2A–2C).^22^

Furthermore, the development of a dual-target MRI nanoprobe, utilizing superparamagnetic iron oxide nanoparticles to specifically label newly formed collateral vessels and inflammatory lesions in the ischemic region of the brain, has provided a new approach for identifying potential hemorrhagic sites in thrombolytic therapy.^74^

The integration of diverse materials into multifunctional magnetic nanoparticles enhances their functionality in MRI, where they act as magnetic contrast agents to augment MRI sensitivity.^75^ The superparamagnetic characteristics of these nanoparticles enable them to function as contrast enhancers, facilitating more precise identification of stroke-induced brain damage.^76^ For instance, a team led by Y.F. at Southeast University devised a magnetic nanoparticle-based carrier utilizing platelet membranes, loaded with L-arginine and γ-Fe_2_O_3_ magnetic nanoparticles. These nanoparticles, coated with platelet membranes, specifically target damaged blood vessels and effectively outline ischemic stroke-induced vascular network injuries under T2∗-weighted MRI. Additionally, the release of L-arginine from these nanoparticles induces vasodilation in occluded vessels, an effect further assessed using DWI, proving to suppress efficiently the expansion of ischemic lesions (Figure 2F).^23^

#### Magnetic particle imaging (MPI)

Superparamagnetic iron oxide nanoparticles (SPIO) can be used as tracers for generating images by detecting their magnetic properties in specific magnetic fields.^77^ Unlike MRI, MPI exclusively visualizes the distribution of tracers, offering high contrast and sensitivity. With superior temporal resolution and a non-radiative nature, MPI facilitates rapid and accurate assessment of brain perfusion, significantly enhancing stroke diagnosis and treatment.^78^ A 2017 study demonstrated that MPI could assess brain perfusion and vascular anatomy in C57BL/6 mice within seconds of SPIO injection, showing performance comparable to MRI.^24^ Furthermore, MPI scanners can be designed as portable, pre-hospital devices that require substantially less infrastructure than CT or MRI, providing a distinct advantage in accelerating stroke diagnosis and treatment.^79^

### Application of nanomaterials in stroke therapy

Nanomaterials have garnered significant interest from the scientific community due to their small size, high surface-to-volume ratio, and ability to be modified on a targeted surface. They hold great potential for use in cerebrovascular accident (stroke) intervention. Nanomaterials possess inherent properties that offer a distinct advantage in pharmacological delivery systems, facilitating the successful crossing of biological barriers, such as the BBB. This opens up new avenues for precision medicine. The application of nanotechnology in stroke management mainly focuses on enhancing therapeutic delivery, neuroprotection, and reducing ischemic reperfusion injury. The ultimate objective is to improve the effectiveness of current stroke treatments and significantly enhance clinical outcomes for stroke patients. This strategic utilization highlights the transformative capabilities of nanotechnology in advancing existing therapeutic approaches.

#### Nanodrug delivery system for treatment

Nanocarriers demonstrate potential as a drug delivery system for treating brain dysfunction. They possess the ability to resist drug degradation, enhance drug metabolism kinetics, and improve neurovascular pathways. Nevertheless, the blood-brain barrier poses a significant challenge in the therapeutic process by restricting drug transportation into the brain. To overcome this obstacle, nanoparticles are modified to enhance the likelihood and concentration of drug delivery to the ischemic site by crossing the blood-brain barrier.^80^ The primary methods for nano-delivery systems to traverse the blood-brain barrier include passive diffusion, adsorption-mediated endocytosis, receptor-mediated transport, and carrier-mediated transport.^81^

##### Thrombolysis therapy

**Figure 3:**
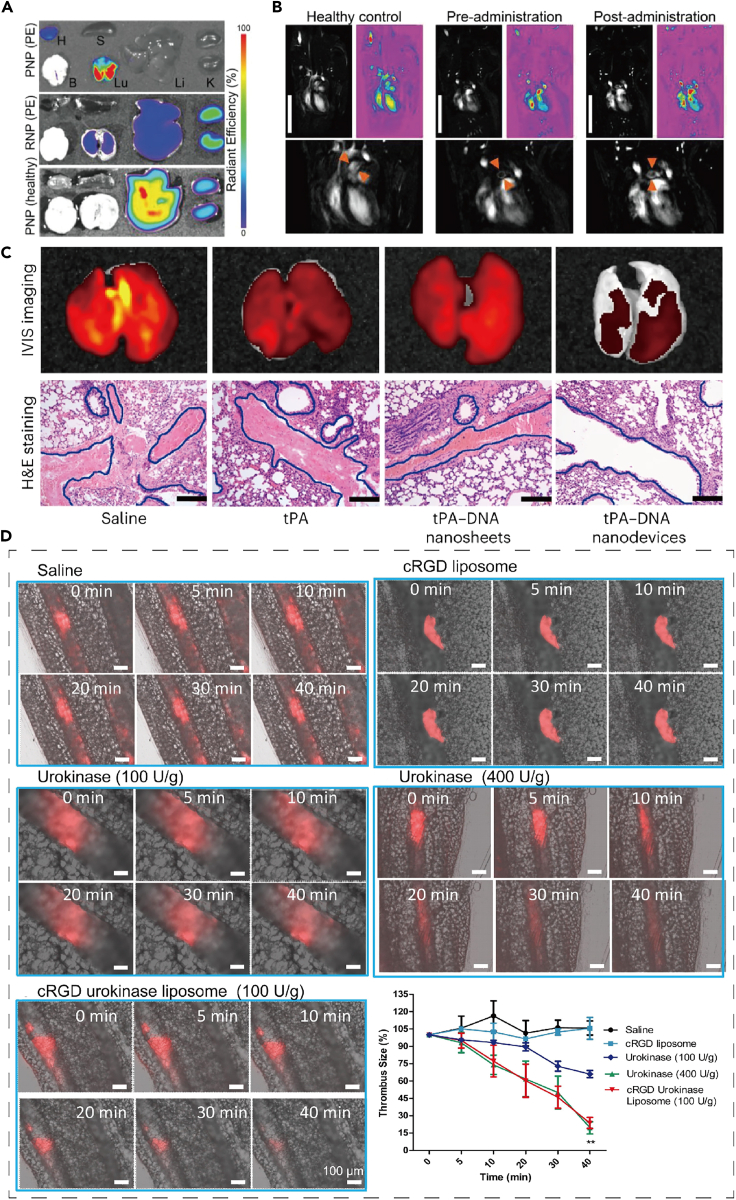
The application of nanoparticles in thrombolysis therapy (A) *Ex vivo* imaging of Cy5.5-labeled PNP-PA in major organs of PE model and healthy mice, 2 h post-injection. H, heart; B, brain; S, spleen; Lu, lung; Li, liver; K, kidney. (*n* = 3). Quantification of the fluorescence levels is shown in the lower panel. (B) T1-weighted MRI of mice before and after PNP-PA treatment. Scale bar: 2 cm. Reproduced with permission from ref.^28^ Copyright 2019 Advanced Materials. (C) Representative fluorescence images (top) and H&E staining images (bottom) of lung tissue from mice treated with saline, tPA, tPA-DNA nanosheets, or tPA-DNA nanodevices. Scale bars: 100 μm. Reproduced with permission from ref.^30^ Copyright 2024 Nature Materials. (D) Real-time thrombolysis results of cRGD liposomes loaded with urokinase in mouse mesenteric vessels were captured at 0, 5, 10, 20, 30, and 40 min after thrombus formation. Reproduced with permission from ref.^27^ Copyright 2018 Acta Biomaterialia.

Encapsulated thrombolytic agents have been developed to deliver the enzyme tissue plasminogen activator (tPA) to the target area at a controllable rate. This helps temporarily inhibit tPA activity in the circulation, extending its half-life and improving its contact with the clot. The goal is to minimize the risk of non-specific bleeding and achieve better efficacy in thrombolysis.^82^^,^^83^^,^^84^ Researchers have also developed silicon-coated magnetic nanoparticles (SiO_2_-MNP) as biocompatible carriers for targeted delivery of tPA.^25^ In addition, SPIONs stabilized by a dextran shell have been created as a magnetite drug carrier for targeted thrombolysis under an applied magnetic field.^26^ Cyclic RGD (cRGD) functionalized liposomes have been employed to encapsulate thrombolytic drugs^27^ (Figure 3D). In 2018, Vankayala et al. identified a nanostructured system that consists of vesicles from erythrocytes encapsulated with near-infrared fluorophores (ICGs) and a tPA affixed to the vesicle surface. This system effectively prolongs the half-life of tPA and reduces the risk of bleeding.^85^ Xu et al. developed platelet membrane-camouflaged polymeric nanoparticles (nanoplatelets) that deliver tPA to localized thrombus sites. In an animal model of thrombosis, these tailored nanoplatelets aggregated efficiently at the thrombus and demonstrated significantly enhanced thrombolytic activity compared to free rt-PA (Figures 3A and 3B).^28^^,^^30^

**Figure 4:**
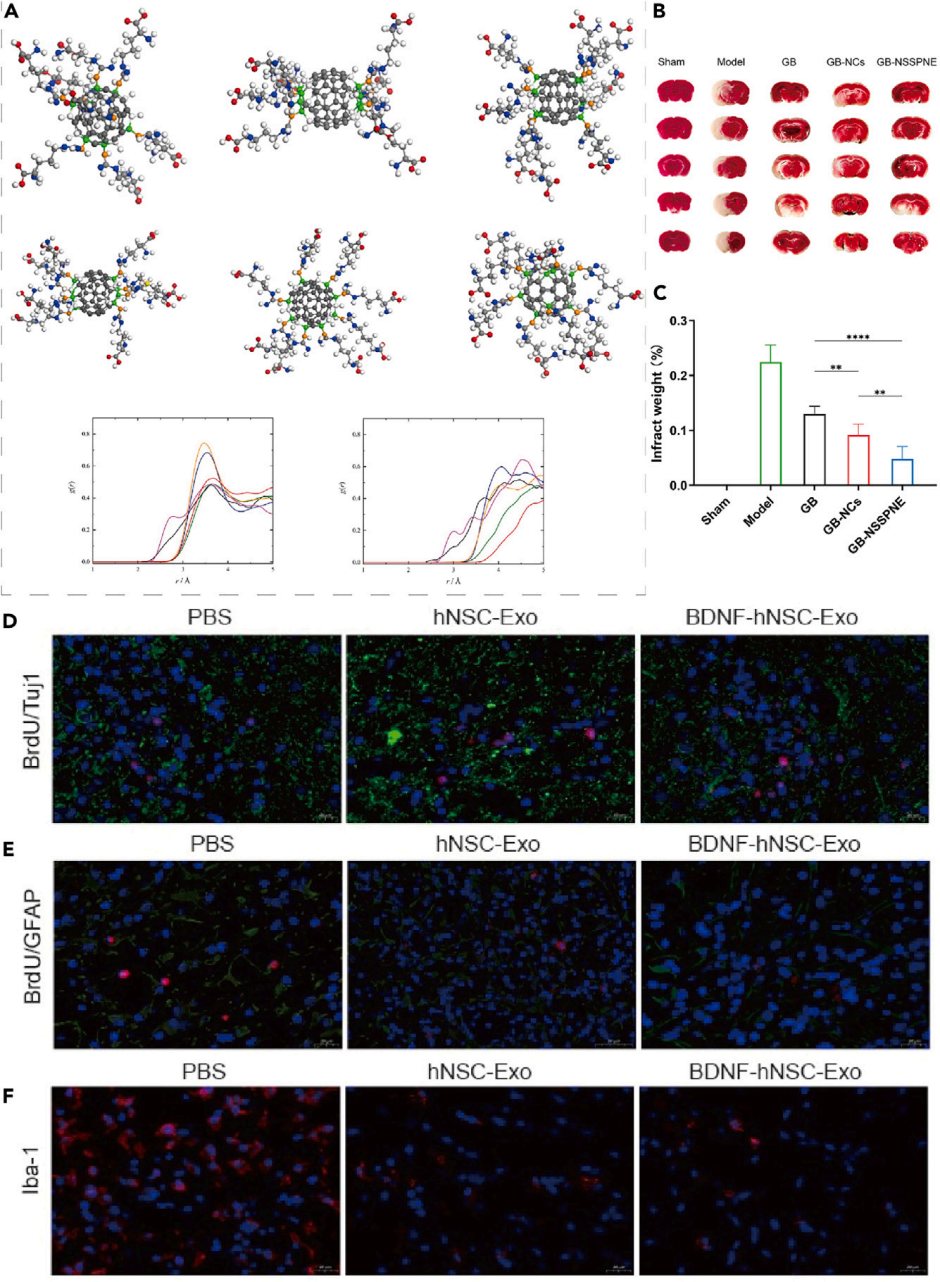
The synergistic effects of nanomaterials on neuroprotection, neurogenesis and reduction of neuroinflammation in ischemic stroke models (A) Water molecules interact with various atoms of the fullerene adduct C60-Arg, with the oxygen atoms distributed around the carbon atoms of the unmodified fullerene core and those bonded to the L-Arg residues. Reproduced with permission from ref.^35^ Copyright 2023 Nanomedicine-Nanotechnology Biology and Medicine. (B) The results of TTC staining. (C) The percentage of cerebral infarct area following ischemia/reperfusion. Date were represented as mean ± SD (*n* = 6/7/8). ∗*p* < 0.05, ∗∗*p* < 0.01, ∗∗∗*p* < 0.001, ∗∗∗∗*p* < 0.0001. Reproduced with permission from ref.^36^ Copyright 2024 European Journal of Pharmaceutical Sciences. (D) Immunofluorescence double labeling showed that more functional neurons were generated in the peri-infarct area in the BDNF-hNSC-Exo and hNSC-Exo groups than in the PBS group. BrdU (red) colocalized with Tuj1 (green). Cell nuclei were stained with DAPI (blue). (E) Double immunofluorescence staining showed that the proportion of BrdU/GFAP double-positive cells in the peri-infarct area was lower in the BDNF-hNSC-Exo group than in the hNSC-Exo group and the PBS group on day 28 after treatment. BrdU (red) colocalized with GFAP (green). Cell nuclei were stained with DAPI (blue). (F) Immunofluorescence showed that BDNF-hNSC-Exo significantly reduced the expression of Iba1 (red), indicating reduced neuroinflammation. Nuclei were stained by DAPI (blue). Scale bar: 20 μm *n* = 5 rats/group. Reproduced with permission from ref.^32^ Copyright 2023 Neural Regeneration Research.

Therapeutic hypothermia is considered a neuroprotective strategy for stroke. Jin et al. have worked on the development of a protein-polymer conjugate (PEG-uPA-PEG-PPG-PEG) that responds to changes in both pH and temperature. This conjugate modifies urokinase-type plasminogen activator (uPA) using polyethylene glycol (PEG) and a polymer known as poly(ethylene glycol)-blocking-poly(propylene glycol)-blocking-poly(ethylene glycol) (PEG-PPG-PEG), which is thermosensitive. By incorporating pH-sensitive bonds like salicylidene and disulfide bonds, the PEG-uPA-PEG-PPG-PEG conjugate remains resistant to protease hydrolysis at normal body temperature and physiological pH, thus masking the bioactivity of uPA and reducing the risk of acute bleeding complications. However, in an acidic pH environment and at a temperature of 33°C, the protein activity is restored, allowing for thrombolytic therapy under mild hypothermia conditions.^29^ In addition, Yin et al. have developed a remarkable DNA thrombolytic nanomachine, which is capable of detecting thrombin, a biomarker for blood clots, in complex pathophysiological conditions within the blood vessels. This nanomachine is also able to distinguish between blood clots and wound clots through logical operations that target the concentration of thrombin, enabling precise delivery of targeted thrombolytics (Figure 3C).^30^

##### Neuroprotective therapy

Preconditioning neural stem cells (NSCs) with hypoxia generate hypoxic exosomes that can regulate the microenvironment of the injured brain. These exosomes exhibit the ability to inhibit neuroinflammation and facilitate the restoration of blood-brain barrier permeability, thus offering a promising approach for effectively treating ischemic stroke.^31^ Additionally, a novel type of exosome, referred to as BDNF-hNSC-Exo, was developed by incorporating brain-derived neurotrophic factor (BDNF) into NSC-derived exosomes. This modified exosome demonstrated a significant enhancement in cell survival when exposed to oxidative stress. Moreover, in an arterial occlusion model, BDNF-hNSC-Exo not only suppressed microglia activation but also facilitated the differentiation of endogenous NSCs into neurons (Figures 4D–4F).^32^

In addition to hypoxia exosomes, nanoparticles can be used to encapsulate hypoxia-inducible factors to protect against nerve damage. Liu et al. successfully encapsulated YC-1, an inhibitor of hypoxia-inducible factor-1α (HIF-1α), within hybrid membrane nanoparticles, known as YC-1@[RBC-PL] NVs, which are made up of red blood cell and platelet membranes. These nanoparticles have shown potential for safeguarding the neurovascular unit (NVU) in cases of ischemic stroke. In a study utilizing the middle cerebral artery occlusion/reperfusion (MCAO/R) model, YC-1@[RBC-PL] NVs demonstrated their ability to reduce ischemic damage to the NVU by decreasing infarct volume, preserving the integrity of the BBB, and inhibiting the activation of astrocytes and microglia.^33^

EVs are a promising form of natural nanomedicines and carriers that can be engineered to improve their application in the treatment of central nervous system diseases. In this study, researchers utilized pro-repair microglia-secreted EVs as a base and modified them by attaching a pro-NSC recruitment signaling molecule, stromal cell-derived factor 1 (SDF-1), to the surface of the EVs through click chemistry. The resulting SDF-1-EVs were found to significantly enhance NSC migration and differentiation into neurons, while also reducing brain atrophy volume. Additionally, the researchers employed microfluidic hydrogel microspheres (MS) as a set of slow-releasing engineered extracellular vesicles (S-EVs@MS) for intracerebral targeted injection to achieve long-term neurorestoration after ischemic stroke.^34^

The water-soluble adduct of fullerene, C60-Arg, is compatible with blood and exhibits significant antioxidant activity. It can cross the blood-brain barrier and release nitric oxide, which is derived from the decomposition of L-arginine residues, thereby promoting vasodilation (Figure 4A).^35^ Ginkgolide B (GB) is a powerful natural antagonist of platelet-activating factor (PAF) and is extensively used in the treatment of cardiovascular diseases. The utilization of self-stabilized Pickering nano-emulsion of GB nanocrystals (GB-NSSPNE), with intact nanoparticles, enables controlled release and enhanced cellular uptake, thereby markedly improving the permeability of GB. As a result, the oral bioavailability of GB is significantly enhanced, leading to the augmentation of its neuroprotective effects (Figures 4B and 4C).^36^

#### Nanoconjugates for protecting the brain from ischemia-reperfusion injury

Ischemia-reperfusion-induced oxidative stress can lead to substantial secondary harm in stroke patients. The impairment of cellular homeostasis caused by ischemia-reperfusion injury (IRI) through apoptosis and autophagy pathways can result in cellular disruption, as well as oxidative stress leading to organ damage through the production of redox free radicals.^86^ This includes excessive ROS that can cause severe harm to cells by damaging organelles, cell membranes, and nuclear DNA.^87^ This includes excessive ROS that can cause severe harm to cells by damaging organelles, cell membranes, and nuclear DNA.

A novel biomimetic drug delivery system, referred to as SHp-NM@Edv/RCD (SNM-NPs), has been developed through recent research to investigate potential therapeutic approaches for cerebral ischemia/reperfusion injury (CIRI). The SNM-NPs system utilizes neutrophil membranes (NMs) to encapsulate the drug, facilitating targeted delivery to the inflammatory microenvironment and exhibiting multi-step targeting capabilities. By modifying the stroke homing peptide (SHp), the SNM-NPs effectively target damaged neurons. In the third step, the drug edaravone (Edv) is precisely released at the site of cerebral ischemia/reperfusion injury by the controlled release device (RCD) enclosed within the SNM-NPs, which is triggered by ROS. This release mechanism effectively scavenges ROS, reduces the number of microglial cells, enhances tubulin expression in neurons, suppresses neuroinflammation, and reduces neuronal apoptosis by 90%, primarily by inhibiting the neuronal Caspase 3 pathway.^37^

A new supramolecular peptide hydrogel, EDV/Cur/NapFFY, has been developed to improve bioavailability and facilitate targeted transport of lipophilic drugs to ischemic sites via local administration. Co-assembly with the well-studied hydrogelator Nap-Phe-Phe-Tyr-OH (NapFFY) was employed in the development process. *In vitro* release tests have demonstrated the sustained release of Cur and Edv over a period of approximately two weeks. Animal studies have further revealed the EDV/Cur/NapFFY hydrogel’s ability to effectively enhance brain plasticity and promote functional recovery in a photothrombotic mouse model. These findings highlight the significant potential of the supramolecular peptide hydrogel delivery system, EDV/Cur/NapFFY, in facilitating repair and recovery from ischemic stroke. This milestone represents a crucial advancement toward more precise and efficacious drug delivery approaches in the treatment of cerebral ischemia.^38^

##### Antioxidant delivery

During the ischemia/reperfusion (I/R) process, the production of hydrogen peroxide (H2O2) can cause inflammation, apoptosis, and tissue damage. To combat this problem, a novel nanotherapeutic agent specifically designed for I/R has been created. This agent consists of antioxidant nanoparticles that are responsive to H2O2 and are formulated with copolyoxyethylene ether (PVAX) containing vanillyl alcohol (VA). Upon degradation of the PVAX nanoparticles, VA is released, which effectively reduces the production of ROS and exhibits both anti-inflammatory and anti-apoptotic effects.^39^

**Figure 5:**
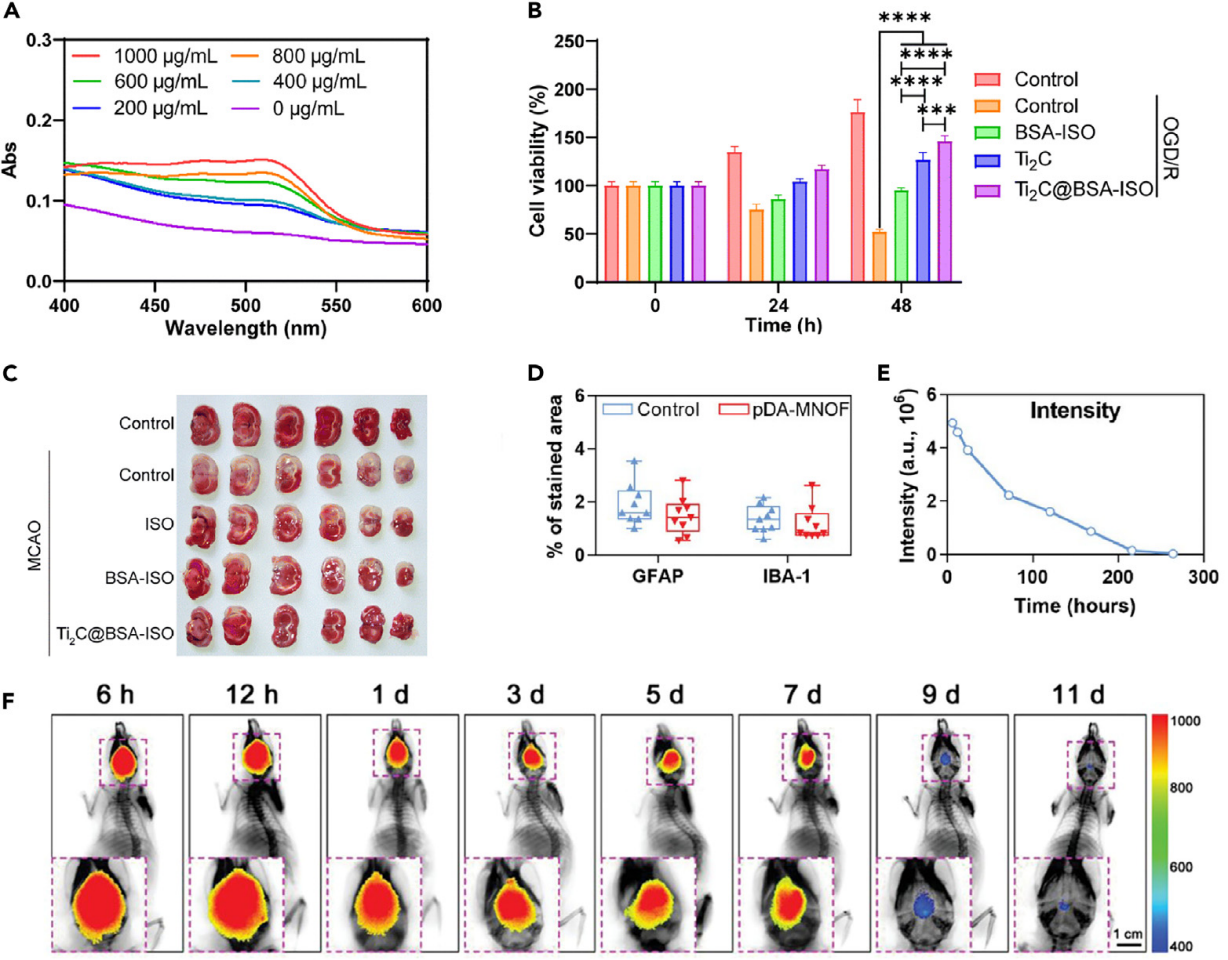
*In vivo* efficacy and biocompatibility of antioxidant delivery materials (A) Total antioxidant capacity of Ti_2_C. The therapeutic effect of Ti_2_C@BSA-ISO *in vitro*. (B) Cell survival of primary hippocampal neurons at 0 h, 24 h, and 48 h in different treatment groups. ∗*p* < 0.05, ∗∗*p* < 0.01, ∗∗∗*p* < 0.001, ∗∗∗∗*p* < 0.0001. (C) Representative images of TTC-stained coronal brain sections of rats after MCAO. Reproduced with permission from ref.^40^ Copyright 2024 Mater Chem. (D) Quantification of the proportion of GFAP-positive and IBA-1-positive regions (*n* = 9 sections). (E) Quantification of fluorescence intensity in the brain at different time points after intracerebral injection of ICG@pDA-MNOF. (F) Representative fluorescence images of mice at different time points after intracerebral injection of ICG@pDA-MNOF. Reproduced with permission from ref.^43^ Copyright 2023 Advanced Science.

Fan et al. developed a nanopreparation for the treatment of ischemic stroke. They used Ti_2_C nanoenzymes, loaded with BSA-encapsulated hydrophobic drug isoquercetin (BSA-ISO). The resulting Ti_2_C@BSA-ISO showed a robust capacity for scavenging free radicals and demonstrated its potential in alleviating ischemic stroke. It achieved this by promoting neuroprotection and scavenging ROS, effectively inhibiting sepsis in both the hippocampal CA1 region and the cerebral cortex of rats (Figures 5A–5C).^40^

Yang et al. developed a nanotherapeutic for ischemic stroke that is responsive to ROS, translatable and triple-targeted with DL-3-*n*-butylphthalide (NBP). This nanotherapeutic aims to reduce endothelial permeability induced by ischemia/reperfusion, improve dendritic remodeling, and enhance synaptic plasticity of neurons in the injured brain tissue. The NBP-loaded SON achieves these effects by effectively delivering NBP to the ischemic brain tissue and targeting injured endothelial cells and activated neurons/microglial cells. Additionally, it normalizes the pathological microenvironment, which may contribute to better functional recovery.^41^ The mechanistic details are provided without subjective evaluations. Resveratrol, an ROS scavenger, exerts neuroprotective effects for the treatment of ischemic stroke by polarizing M1 microglia to an anti-inflammatory M2 phenotype. A nano platform composed of pH-responsive poly(ethylene glycol)-acetal-polycaprolactone-poly(ethylene glycol) (PEG-Acetal-PCL-PEG) and modified with cRGD and TPP on the long and short PEG chains, respectively, effectively alleviates oxidative stress and inflammation. This is achieved by enhancing resveratrol delivery to microglia mitochondria and scavenging ROS to reverse microglia phenotype.^42^ The pDA-MNOF nanoparticle, based on a manganese organic framework, mimics the catalytic activity of natural dismutase. It up-regulates the expression of two endogenous antioxidant enzymes, HO1 and SOD2, in neuronal cells through the STAT3 signaling pathway. This effectively protects neurons from oxidative stress damage, reduces the volume of cerebral infarction, and promotes the recovery of behavioral function through the dual intracellular and extracellular scavenging and pro-angiogenic effects of free radicals. The recovery of behavioral functions has been observed (Figures 5D–5F).^43^

##### Anti-inflammatory agent

**Figure 6:**
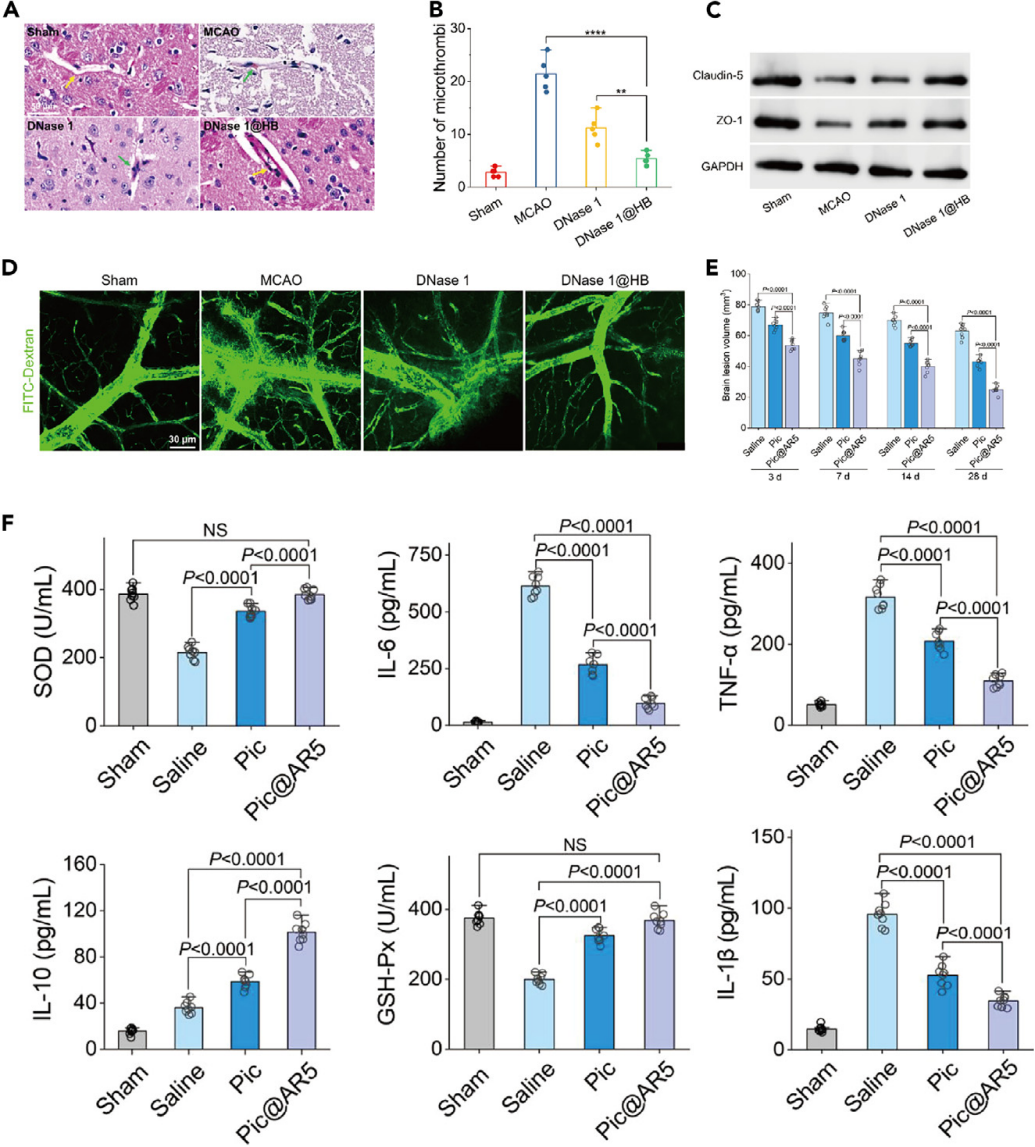
Nanoparticles enhance drug delivery and alleviate neuroinflammation (A–D) Treatment of 1@HB with DNase inhibited MCAO-induced microvascular thrombosis and reduced the number of microthrombi, improved microvascular patency and avoided vascular leakage. ∗*p* < 0.05, ∗∗*p* < 0.01, ∗∗∗*p* < 0.001, ∗∗∗∗*p* < 0.0001. Reproduced with permission from ref.^44^ Copyright 2024 Nano Today. (E and F) AR5 nanoparticles enhance leupeptin delivery to the inflamed brain and alleviate neuroinflammation Reproduced with permission from ref.^45^ Copyright 2023 Advanced Functional Materials.

Neutrophils play a significant role in the pathophysiology of ischemic stroke due to their association with pro-inflammatory processes. A potential strategy for treating this condition involves preventing the infiltration of neutrophils into ischemic areas. A pH-triggered polymer nanoplatform has been developed to enhance the delivery of DNase 1 to the brain, offering relief from ischemic stroke-related thrombotic inflammation. This polymer is designed to disintegrate in an acidic microenvironment, resulting in the release of DNase 1. The enzyme is then able to scavenge neutrophil extracellular traps (NETs), thereby disrupting the structure of the thrombus backbone and inhibiting microthrombosis. Additionally, DNase 1 destabilizes histone proteins in NETs and prevents platelet activation through the TLR4 pathway. This, in turn, downregulates HMGB-1, thus avoiding NET formation. By breaking down NETs and inhibiting platelet activation, both platelet and neutrophil aggregation can be effectively reduced (Figures 6A–6D).^44^ Recent studies have demonstrated that nanoparticles with an aspect ratio (AR) of 5 loaded with piceatannol (Pic@AR5) can hinder the adhesion of neutrophils to endothelial cells, thus preventing their infiltration into the BBB. Furthermore, even if only a small number of neutrophils carrying Pic@AR5 manage to enter the BBB, the inflammatory cytokines in the ischemic brain can still be reduced. This is because the nanoparticles release piceatannol within the ischemic region, which inhibits microglial Syk signaling and leads to the alleviation of neuroinflammation (Figures 6E and 6F)).^45^

After an ischemic stroke, damaged tissue releases cell-free double-stranded DNA (dsDNA) fragments, which microglia phagocytose. The cyclic GMP-AMP synthase (cGAS) of microglia recognizes these fragments, activating the stimulator of the interferon genes (STING) pathway. Consequently, a chronic inflammatory response ensues. To address this, researchers have developed a nanomedicine loaded with the C-176 drug. The nanomedicine named NTA/Ce4+/C-176 utilizes amphiphilic copolymers (P[CL35-b-(OEGMA20.7-co-NTAMA14.3)]), C-176, and Ce4+ coordinated with nitrilotriacetic acid (NTA) to self-assemble into nanoparticles. This innovative approach combines the anti-inflammatory strategies of the DNase-mimetic Ce4+ enzyme and STING inhibitors. The primary goal of this nanomedicine is to restore balance to the cGAS-STING axis, enhancing stroke treatment.^46^

The drug-free biomimetic nanovehicle has two main components: an amphiphilic polyfluorocarbon that delivers oxygen to ischemic brain regions to alleviate the hypoxic environment, and Pluronic P123, which inhibits matrix metalloproteinase-9 (MMP-9) to protect the BBB. When administered intravenously to rats with transient middle cerebral artery occlusion caused by ischemic stroke, these nano vehicles can specifically target cerebral ischemic lesions in an inflammation-directed manner. This approach provides robust tolerance to ischemia and resistance to reperfusion for ischemia-reperfusion therapy.^47^

The OMV@PGZ nanosystem, consisting of pioglitazone (PGZ) nanoparticles encapsulated by bacterial-derived outer-membrane vesicles (OMVs), effectively crosses the blood-brain barrier and accumulates in ischemic areas, where it suppresses ferroptosis and reduces inflammation by releasing PGZ.^48^ Another study highlighted a nanosystem’s ability to utilize blood neutrophils for targeted delivery. The surface of the nanosystem is modified with the peptide CFLFLF, which binds to the FPR on neutrophils, allowing the nanosystem to harness the migratory capabilities of these immune cells to reach ischemic brain regions.^49^

#### Gene therapy

In gene therapy for stroke, nanomaterials are used for precise delivery of therapeutic genes, enhancing gene transfection efficiency, protecting genes from degradation, and enabling targeted, effective treatment of brain tissue. In the context of cerebral infarction, the affected region demonstrates overexpression of the long non-coding RNA (lncRNA) known as maternally expressed gene 3 (Meg3). Suppression of Meg3 has been associated with enhanced neovascularization and improved neurological function. This study proposes an innovative therapeutic approach that utilizes a nanopolymer to encapsulate a Meg3-targeting short hairpin RNA (shRNA) plasmid, to achieve precise Meg3 knockdown. Additionally, the nanopolymer is conjugated with the OX26 antibody (MPO) to enhance brain-targeted delivery. This strategy holds significant potential in promoting angiogenesis and facilitating neurological recovery, thus offering an advanced treatment option for individuals with cerebral infarction.^50^

Jiang et al. developed a charge-reversal polymer called poly[(2-acryloyl) ethyl (p-boronic acid benzyl) diethylammonium bromide] (B-PDEA) vector, which is responsive to ROS. This vector was used to efficiently mediate gene transfection in NSCs. As a result, it successfully induced the expression of BDNF and showed high therapeutic efficacy in the treatment of ischemic stroke.^51^ Additionally, the use of poly-lactic-co-glycolic acid (PLGA) nanoparticles was found to enhance the expression of the SOX9 gene in GFAP-positive astrocytes. PLGA nanoparticles encapsulated with GFAP:SOX9:tdTOM reduce ischemia-induced neurological deficits and infarct volume via the prostaglandin D2 pathway. This finding highlights the significant role of nanoparticle-mediated gene delivery in modulating astrocyte function and mitigating ischemic brain injury.^52^

Magnetic cell guidance is achieved by transfecting neural stem cells with zinc-doped ferrite magnetic nanoparticles (ZnMNPs), enabling these cells to migrate to targeted brain regions under an external magnetic field. The mechanical forces exerted by the ZnMNPs can overcome spatial barriers within brain tissue, facilitating directed migration. At the lesion site, the magnetically labeled cells (Mag-Cells) demonstrate enhanced neuronal differentiation and secrete significantly more neurotrophic factors compared to unlabeled control cells. This effect is mediated by ZnMNPs, which activate zinc-induced Wnt signaling, promoting neuronal differentiation. In a rodent stroke model, Mag-Cells significantly improved motor function recovery in the impaired limbs.^53^

### Biomimetic nanomaterials

Biomimetic nanomaterials, designed and engineered at the nanoscale to replicate natural structures, functions, or processes, hold significant potential in biomedical applications including drug delivery, tissue engineering, diagnostic imaging, and therapy.^88^

Applications in ischemic stroke treatment primarily focus on drug delivery and microenvironment regulation. Professor Jiang Chen’s team has developed a bioinspired nanoerythrocyte, CPTK@PMH, tailored for the comprehensive management of AIS. This nanoerythrocyte remodels the metabolic microenvironment to improve long-term prognosis. It targets microthrombi, navigates effectively to the ischemic core, and enhances drug accumulation. During the ischemic phase, it releases oxygen to mitigate hypoxia. In the reperfusion phase, it captures excess oxygen to prevent ROS production and supports microglial polarization. In the recovery phase, it dispenses drugs that stimulate glucose metabolism and safeguard the blood-brain barrier, thus offering a multi-targeted, multilevel treatment approach for AIS.^54^

A novel nano-drug delivery platform has been developed, capable of crossing the blood-brain barrier to specifically target ischemic stroke lesions. Through the innovative use of 4T1 breast cancer cell membranes and platelet membranes, nanocarriers encapsulating paeonol and polymet liposomes (PP@PCL) have been engineered. These carriers have proven effective in inhibiting neuroinflammation, increasing cerebrovascular density, promoting neurovascular regeneration, and remodeling the ischemic microenvironment.^55^ To enhance therapeutic strategies for ischemic stroke further, a groundbreaking approach that focuses on boosting the efficacy of stem cell therapies has been introduced. A biomimetic ferrimagnetic iron oxide nanochain (MFION) was developed to enhance the efficiency and safety of gene recombination in mesenchymal stem cells (MSCs). This innovation is particularly significant as it markedly increases BDNF secretion in human placental-derived MSCs and upregulates CXCR4 expression, thus improving the MSCs’ homing capabilities to ischemic brain tissue post tail vein injection. Consequently, in a murine ischemic stroke model, MFION-modified MSCs showed improved survival and expedited neuronal recovery, surpassing conventional MSC-based treatments.^56^

These studies illustrate that biomimetic nanomaterials possess substantial potential to enhance drug delivery, improve therapeutic outcomes, and facilitate recovery post-stroke. Through meticulous design, these nanoplatforms can specifically target the multifaceted pathophysiological mechanisms of ischemic stroke, offering more efficient and targeted therapeutic solutions.

### Rescuing organelles and promoting angiogenesis

A ceria nanoenzyme-based synergistic drug-carrying nanosystem targeting mitochondria has been developed to address multiple aspects of ischemic stroke. This nanosystem functions through its individual components and their synergistic interactions, delivering comprehensive therapy. It alleviates oxidative stress and modulates the mitochondrial microenvironment toward a state favorable for ischemic tolerance, thereby restoring the ischemic microenvironment and addressing mitochondrial and other related injuries. Moreover, this study elucidates the detailed mechanisms by which the proposed nanodelivery system protects the brain, signifying a paradigm shift in ischemic stroke treatment.^89^

Similarly, another nanoparticle-based drug delivery platform has shown substantial effectiveness in protecting against ischemic stroke through both *in vivo* and *in vitro* experiments. This system functions by scavenging ROS, mitigating mitochondrial dysfunction, and curbing neuroinflammation. Additionally, it induces polarization of microglia toward the M2 phenotype, enhances angiogenesis, and diminishes pro-inflammatory cytokines, thus transforming the ischemic microenvironment and extending survival in rats. By crossing the blood-brain barrier and specifically targeting lesions, this platform integrates Danshen and Polymet to induce a synergistic effect that bolsters mitochondrial protection and neurovascular regeneration.^89^

Researchers have developed a transferrin receptor (TfR)-targeted nanocarrier (PATRC) aimed at enhancing penetration of the blood-brain barrier (BBB) for cerebral infarction treatment. Compared to using Rg1 alone, PATRC significantly improved tube formation *in vitro*. *In vivo*, it successfully crossed the BBB, reducing cerebral infarct volume and promoting microvascular regeneration in the affected area, highlighting its potential for extensive clinical application.^57^ Expanding on this methodology, another study proposes a dual-target strategy to further refine stroke treatment through pH-sensitive drug release and targeted action on cerebral ischemia. This strategy involves attaching Pro-His-Ser-Arg-Asn (PHSRN) peptides to nanoparticles, which specifically bind to integrin α_5_β_1_ receptors in ischemic brain tissues. Additionally, a smoothened agonist (SAG), an activator of sonic hedgehog (Shh) signaling, is linked to these nanoparticles through pH-dependent adsorption, enhancing drug release in the acidic ischemic environment. The synergistic effect of PHSRN and SAG promotes angiogenesis and strengthens the BBB, leading to improved neuroplasticity and enhanced recovery of neurological functions.^58^

### Toxicity and biocompatibility of nanomaterials

Nanomaterials have shown significant biological effects, but have also raised serious safety concerns. The cytotoxicity of carbon-based nanomaterials is primarily demonstrated through ROS generation, DNA damage, lysosomal and mitochondrial dysfunction, and ultimately apoptosis or necrosis.^90^ Likewise, metal nanoparticles, with their unique physicochemical properties, pose an unforeseen risk of toxicity to the human body, despite offering new opportunities for biomedical research.^91^ The physicochemical properties of nanoparticles, such as surface composition, charge, size, and shape, play a crucial role in determining their biocompatibility and cellular uptake efficiency.^92^ Understanding these properties is essential in evaluating the safety and effectiveness of nanomaterials. Fortunately, modifying the properties of nanoparticles, particularly through surface functionalization techniques, can significantly improve their biocompatibility and intracellular uptake efficiency. This surface modification not only reduces the toxicity risk of nanoparticles but also enhances their potential for clinical applications, making them a powerful tool in medical fields like diagnostics, therapy, and drug delivery.

### Conclusion and outlook

Nanomaterials and nanotechnology are emerging as transformative elements in the field of stroke diagnosis and treatment, offering an optimistic outlook for advancements. In diagnostic applications, nanomaterials significantly enhance the precision of early stroke detection due to their superior sensitivity and specificity. Functionalized nanoparticles, such as PLGA nanoparticles, ROS-responsive polymers, and magnetic nanoparticles, improve the contrast in imaging techniques like MRI and CT. This enhancement aids in the precise localization and identification of stroke lesions, establishing a robust foundation for timely and effective therapeutic interventions and potentially elevating the success of clinical treatments. In the treatment arena, the benefits of nanomaterials are even more pronounced. Their capability for targeted drug delivery allows therapeutic agents to be accurately directed to areas of the brain affected by stroke, significantly increasing drug concentrations at the target site while concurrently minimizing systemic side effects. Moreover, nanomaterials' impressive efficacy in promoting angiogenesis, mitigating neuroinflammation, and repairing damaged tissues paves the way for innovative treatment avenues. These advancements not only bolster therapeutic outcomes but also offer new hope for enhancing patient recovery and quality of life post-stroke. Despite the promise, the path to clinical application of nanotechnology is fraught with challenges. Concerns regarding biocompatibility, long-term safety, and standardization of manufacturing processes necessitate thorough clinical trials and continuous research.

Looking ahead, the application of nanotechnology in both the treatment and diagnosis of stroke is poised to undergo a groundbreaking phase of development. As technological advancements continue and our understanding of stroke pathogenesis deepens, we can anticipate the emergence of more sophisticated nanotherapeutic strategies. These strategies are set to transcend conventional applications such as targeted drug delivery and imaging enhancements, and will encompass gene therapy, neuroprotection, inflammation modulation, among other areas, forging a comprehensive, multi-faceted treatment paradigm. In the realm of gene therapy, nanomaterials are expected to function as highly efficient gene carriers, precisely delivering therapeutic genes to damaged brain tissues and facilitating the expression of neuroprotective factors. This action is poised to expedite nerve repair and regeneration. Concurrently, nanoparticle-based targeted drug delivery systems will undergo further refinement to more effectively traverse the blood-brain barrier, ensuring precise drug distribution and sustained release within the brain, thus enhancing therapeutic efficacy while minimizing adverse effects. In terms of diagnostic imaging, advanced nanocontrast agents will enhance image clarity and resolution, enabling more precise assessments of stroke severity and the extent of lesions. These improvements will robustly support the development of tailored treatment plans based on individual patient profiles. Additionally, nanotechnology is set to integrate seamlessly with cutting-edge technologies such as artificial intelligence and big data, driving the future of stroke diagnosis and treatment toward enhanced intelligence and precision. Achieving these ambitious goals will require robust interdisciplinary collaboration. Cross-disciplinary research that includes medicine, materials science, bioengineering, and computer science will catalyze innovative solutions for the clinical application of nanomaterials and nanotechnologies in stroke treatment. This collaborative effort will significantly advance personalized medicine, allowing for the customization of optimal treatment plans tailored to individual patients. As nanotechnology continues to evolve and enhance its capabilities, the prospects for recovery and quality of life improvement in stroke patients will markedly increase.

## Acknowledgments

This work was supported by the Excellent Young Science Fund for the 10.13039/501100001809National Natural Science Foundation of China (82022033), Sichuan Provincial Science and Technology Program (2024NSFJQ0048), 10.13039/501100001809China National Natural Science Foundation (No. 81902422), 10.13039/501100004608Jiangsu Natural Science Foundation (No. BK20231245), Program of Jiangsu Commission of Health (No. M2020024), Program of Yangzhou Commission of Health (No. 2023-2-01, 2024-2-08).

## Author contributions

Y.L. and J.L. are the co-first authors of this review. K.Z., B.Y., and X.Y. proposed this theme, organized the skeleton, provided the raw materials, and selected the cited references. K.Z., B.Y., X.Y., Y.L., J.L., H.G., C.F., Q.Y., W.Q., H.W., and Y.X. re-organized figures and wrote the manuscript. K.Z., B.Y., and X.Y. revised this manuscript, and supervised and supported the project. All authors commented on this manuscript.

## Declaration of interests

The authors declare no competing financial interest.
